# INTERPLAST-Germany—adapting to global plastic surgery

**DOI:** 10.1007/s00238-023-02051-7

**Published:** 2023-02-15

**Authors:** Andre´ Borsche

**Affiliations:** Department of Plastic and Reconstructive Surgery, Diakonie-Krankenhaus, Ringstr. 64, 55543 Bad Kreuznach, Germany

**Keywords:** INTERPLAST-Germany, Developing countries, Facial reconstruction, Humanitarian missions

## Abstract

**Background:**

INTERPLAST, Inc. was founded in 1969 by plastic surgeons in Stanford, CA, to create a financial basis through donations to operate foreign patients at Stanford or send surgical teams to developing countries. With the same financial effort, 50 to 100 times more patients can be operated on locally than in a hospital at home. Stanford’s example was appealing to many plastic surgeons worldwide, who founded similar INTERPLAST NGOs in their own countries.

**Methods:**

A literature review revealed worldwide humanitarian actions of INTERPLAST teams, whose annual effectiveness is comparable to the operation numbers of large plastic surgery departments. Six patients with complex facial deformities requiring multiple surgical interventions were selected for temporary stay and operations in Germany.

**Results:**

Repeated missions at the same hospitals with training of local surgeons have increased significantly compared to earlier “parachute missions.” Microsurgical procedures for free flaps with magnifying glasses are now possible for the experienced even in hospitals in developing countries. The most efficient medical aid in the future will be the expansion and establishment of departments or hospitals in developing countries supported or maintained by partner hospitals in our home country.

**Conclusions:**

Operations of humanitarian plastic surgeons in developing countries are becoming increasingly difficult. Local health authorities require temporary surgical permits, customs offices try to clear surgical material and look at expiration dates of medicines, and pandemics complicate planning of INTERPLAST missions. It therefore seems increasingly necessary to go as single teacher, training engaged local surgeons and assisting operations. The alternative is inviting local surgeons to Western hospitals and introducing them to the basic skills of plastic surgery and empathy with the poor. However, the lack of recognition of medical degrees from all developing countries remains a problem for their training in Europe.

Level of evidence: Level V, risk/prognostic

## Introduction

Plastic surgery has the advantage over other surgical specialties that it requires few diagnostic tools—and thus provides all prerequisites for operating under simple conditions in developing countries. The model for the creation of INTERPLAST-Germany, e.V. was INTERPLAST Inc., USA, founded in 1969 by Donald Laub, Head of Plastic Surgery at Stanford University, CA [1]. It was common practice to operate patients from developing countries in the USA, but with increasing costs of hospitalization, it became increasingly difficult to find private financiers. Implementing an NGO at Stanford allowed prospective sponsors to receive donation receipts for tax deductions.

### Achievements in the past 40 years

INTERPLAST Inc., USA was renamed “ReSurge International” in 2010 [1] to distinguish itself from 10 other INTERPLAST organizations, which had since been founded worldwide according to the original US model. INTERPLAST-Germany was founded in 1980 [2], INTERPLAST-Australia in1983 [3, 4], INTERPLAST-UK in 1986 [5], INTERPLAST-Turkey in 1988 [6, 7], INTERPLAST-Italy in 1990 [8], INTERPLAST-France in 1990 [9–11], INTERPLAST-Holland in 1996 [12], INTERPLAST-Belgium in 2008, INTERPLAST-Switzerland in 2008, INTERPLAST-Hungary in 2012, and INTERPLAST-India in 2012.

**Fig. 1:**
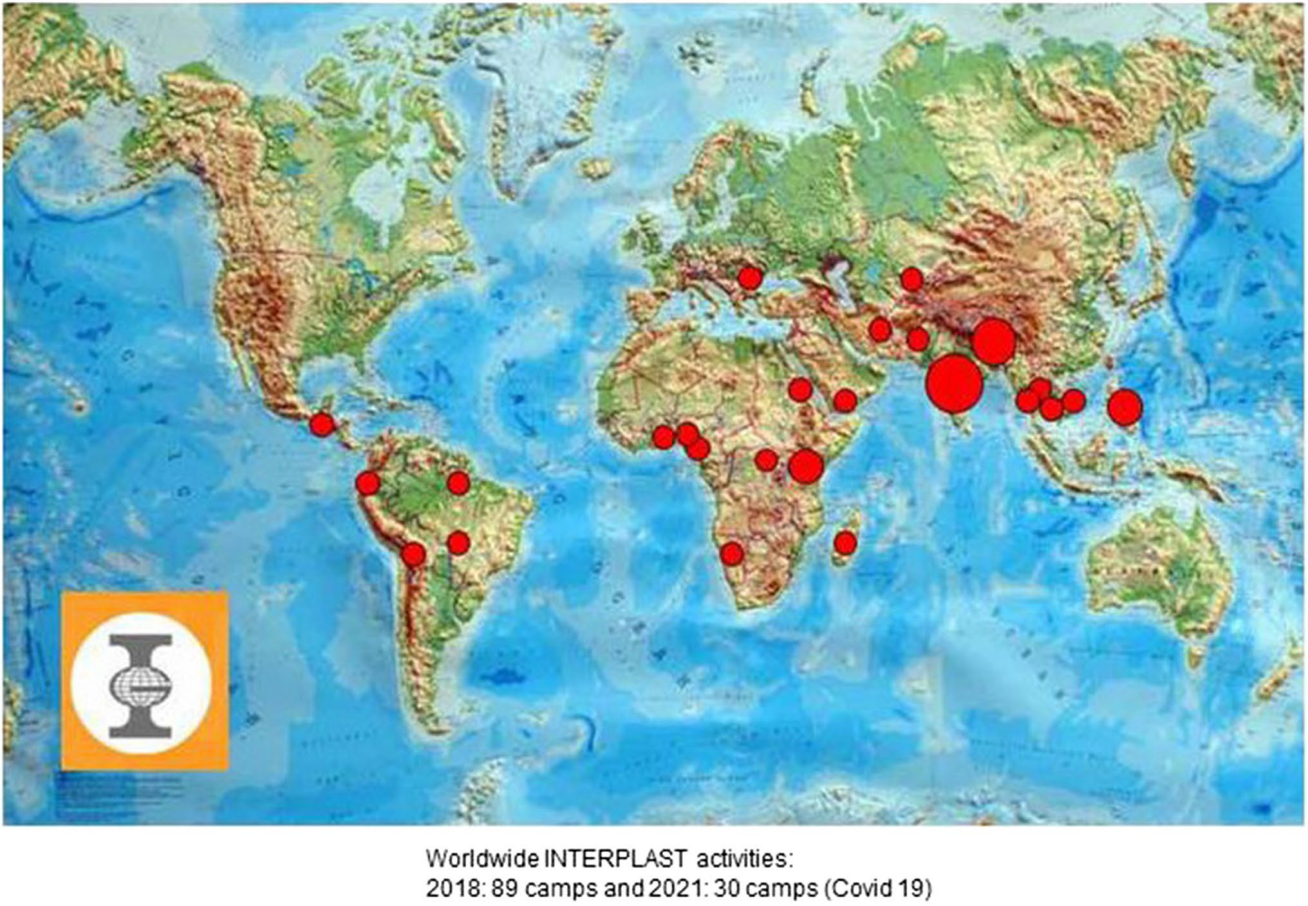
Thirty INTERPLAST-Germany teams organized missions to their partner hospitals worldwide in 2021 despite COVID-19 pandemic

**Fig. 2:**
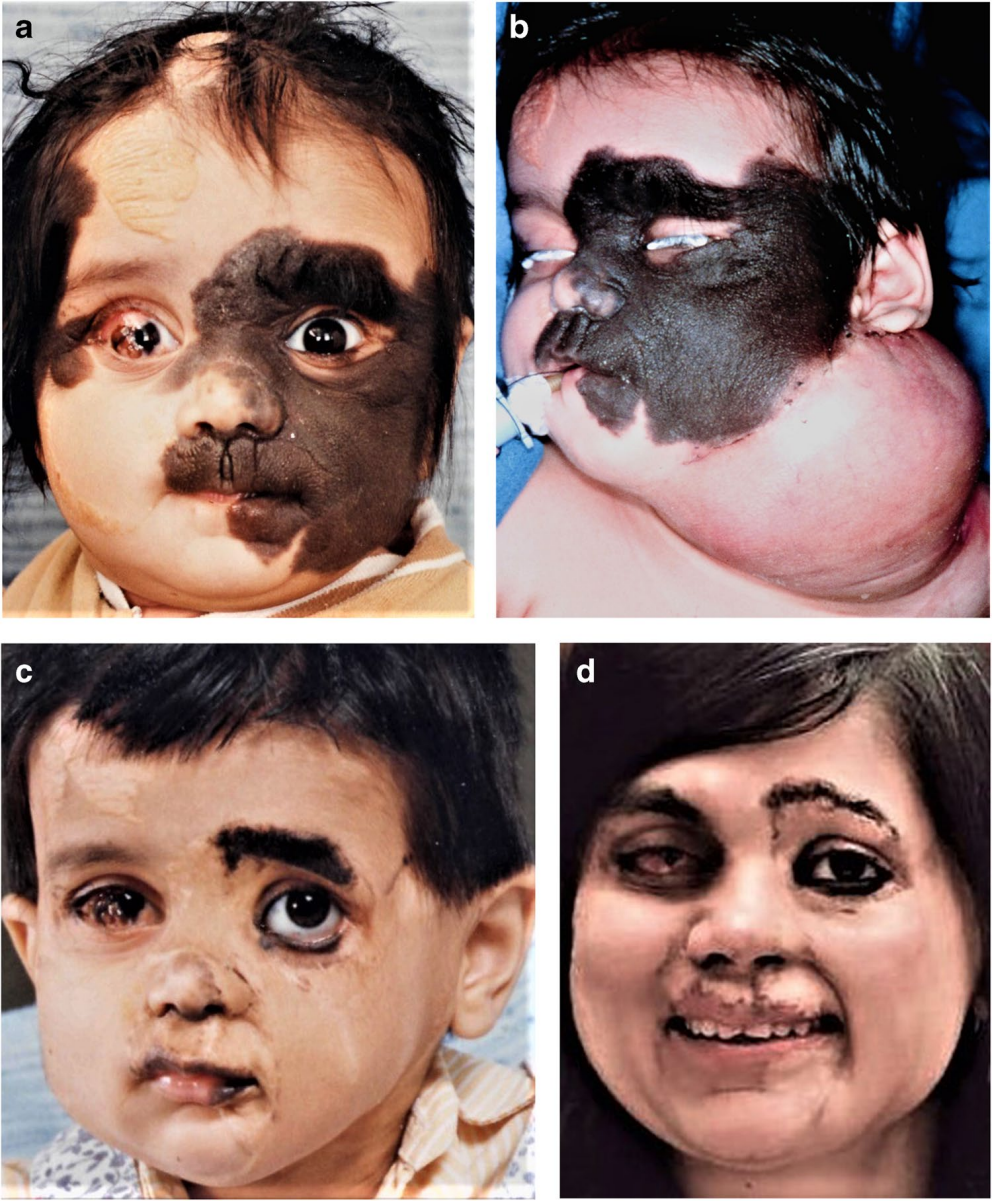
**A** At the airport in Mumbai, this 2-year-old girl with a giant fur nevus on her face was introduced to the German team and treatment in Germany was arranged. **b** A neck expander was slowly filled to cover the largest area of the left cheek with normally perfused skin. **c** The remaining areas were treated with full-thickness skin grafts. After several weeks, the happy family was able to return to India. **d** The self-confident law student now lives happily in Australia

Some INTERPLAST organizations are structured completely central, such as ReSurge International [1]; others were decentralized and self-determining, such as the 14 subsections of INTERPLAST-Germany (Fig. 1) [13, 14]. Today, its > 2400 members include > 370 plastic surgeons, > 350 anesthetists, > 500 other specialties, > 600 nurses, and > 400 sponsors Through this NGO for humanitarian aid, “surgery camps” are organized in most developing countries and very difficult or time-consuming cases are flown for surgery into Germany (Fig. 2a–d).

**Fig. 3:**
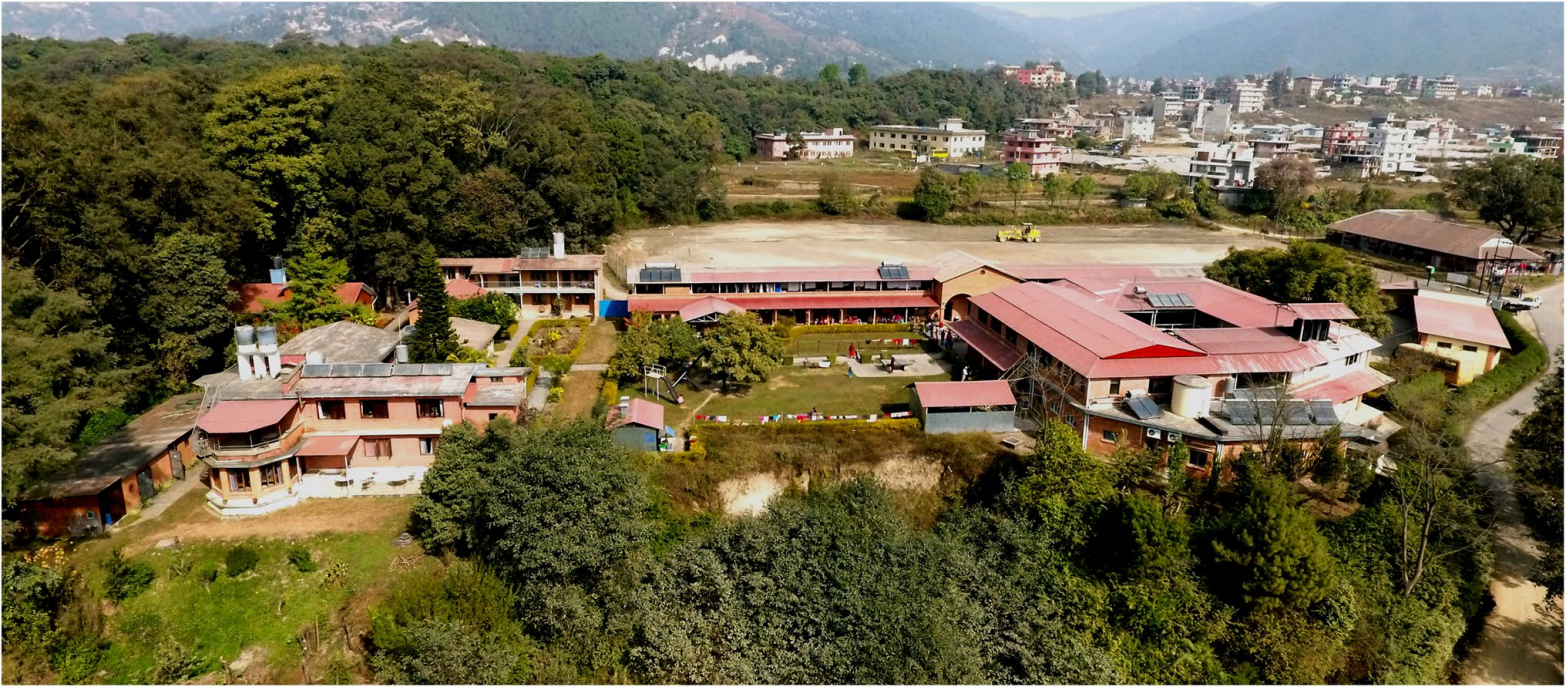
The second INTERPLAST hospital, the Sushma Koirala Memorial Hospital, 18 km east of Kathmandu, Nepal, built since 1997 by the former German Air Force general Hein Stahl

In the last 40 years, more than 1500 INTERPLAST-Germany teams have operated on more than 100,000 patients and are overseeing 4 hospitals in Afghanistan [15], Nepal (Fig. 3) [16–18], Brazil, and Congo [19, 20]. The 14 sections function independently; they are responsible for their own fundraising and the organization of different projects and “surgery camps” in the various developing countries; they use the mother organization mainly for donation and tax deduction receipts and for insurance purposes.

### Costs of INTERPLAST missions

A ReSurge International Team [21] analyzed the costs of 22 surgical trips to 8 different developing countries with 756 surgical interventions between 2014 and 2017. ReSurge paid for the 22 surgical trips a total of $9,795,384 divided by 756 operations = $13,000 per operation. In 2020 during the Corona pandemic, 21 INTERPLAST-Germany teams spent a total of $587,769 divided by 835 operated patients which resulted in $704 per patient. In 2019 before Corona, 79 teams spent $1,252,281 for the treatment of 3338 patients, which means $375 per patient. ReSurge teams are put together by a central office and usually consist of 10–20 professionals, rarely including local colleagues, while the 16 INTERPLAST-Germany sections have to beg for their own expenses and therefore limit their teams to 4–8 members and rely on the local staff.

Another American NGO, “Operation Smile,” focused on cleft lip and palate operations analyzed the cost per patient during eight Operation Smile missions with $280 and $1800 with an average of $800. Using the life tables modified by the author and founder Bill Magee, the cost per patient was between $7.40 and $96.00 (average $34.00) [22]. From their yearly $100 million budget, they can pay local surgeons $240 to $400 per operated cleft patient and just trained 33 surgeons in Rwanda, who earn with one cleft operation the monthly salary of their nurse. Clefts have become a business, unfortunately. INTERPLAST never pays anybody except its secretary in Bad Kreuznach.

### What has changed in the developing countries?

Plastic surgery has entered most developing countries worldwide, and local surgeons are increasingly aware of the benefits of our specialty. The Internet and YouTube enable communication with local surgeons and their education: they can send their complex cases via e-mail or even Zoom and get our virtual support.

On the other side, political and national self-confidence increased significantly, often resulting in rejection of help from outside. A new fear of postcolonialism by rich countries has spread particularly in developing countries with a frightening history, for example, the Congo under the Belgian King. India, Brazil, and Mexico have enough plastic surgeons to serve their underprivileged citizens but often refuse treatment for free, unfortunately.

Politicians and customs authorities are increasingly setting up bureaucratic hurdles to entries and work permits, and modern financial pressure from hospitals sometimes impacts our working conditions. They suddenly ask for recruiting patients and operating room costs, expect expense allowance, and have other monetary demands. As a rule, each mission is a joint venture, and INTERPLAST finds itself permanently in the guest role.

## INTERPLAST activities and possibilities to help

### Modern global surgery

Delivery of surgical care is a crucial component of a properly functioning healthcare system and a prerequisite for universal health coverage. Today, an estimated two billion people worldwide are without adequate access to surgical care.

### Sending INTERPLAST teams to developing countries

As described above, the “standard of care” are still INTERLAST teams not sent but invited by hospitals in developing countries with the goal of a continuing partnership. The knowledge that the team returns every year facilitates the organization and gives hope to patients who missed the present team. Furthermore, the team knows what the hospital needs and which instruments or appliances or devices have to be brought the next time for special patient’s need.

During its 25 years of existence, the Bad Kreuznach INTERPLAST section organized 210 missions to India, Bangladesh, Nepal, Iran, Syria, Tanzania, Ruanda, Cameroon, Brazil, Peru, Bolivia, and most importantly to Haiti following the earthquake in 2010 and to Grozny, Chechnya, after the war in 2006–2008 [23].

The present COVID-19 pandemic reduced our INTERPLAST activities by half, i.e., from 75 missions in 2020 to 30 missions in 2021. Reasons were the curtailment of global travel activities, team members’ self-hazard, possible infection introduction into host countries, and uncertainty about the vaccination status of on-site staff. We hope for a full resumption of previous deployment activities in 2023. There is no increase in use of online resources to connect with host countries during the pandemic.

While in Asia, we look to the future with a good face: when we have a lip or palate operation, 3 to 5 young surgeons stand behind us and record the operation; when we come next time, they have already operated on all the simple crevices. In many places in Africa, unfortunately, it looks different. On the first day, we have 10 visitors in the operating room, on the 2nd still 2, and on the 3rd day rarely a colleague is interested in our specialty. Why shall they learn to do the operations themselves when they can expect us to come again? In Asia, global surgery is clear in its intent to empower LMIC to provide their own care and teach their own surgeons, not to unburden them intermittently.

### Operations on foreign patients in Europe

Due to the high costs, only selected patients can be brought to Europe for surgery. The reasons are mostly lack of diagnostics and expertise on site, a safety risk with local anesthesia, especially in Africa, where mostly nurses or clinical officers do the anesthesia. In addition, there is a high surgical risk with complex malformations and necessary repeat operations, as well as a lack of rehabilitation options and prosthetic care. Uncertain local political conditions in certain countries prevent the sending of a team sometimes.

**Fig. 4:**
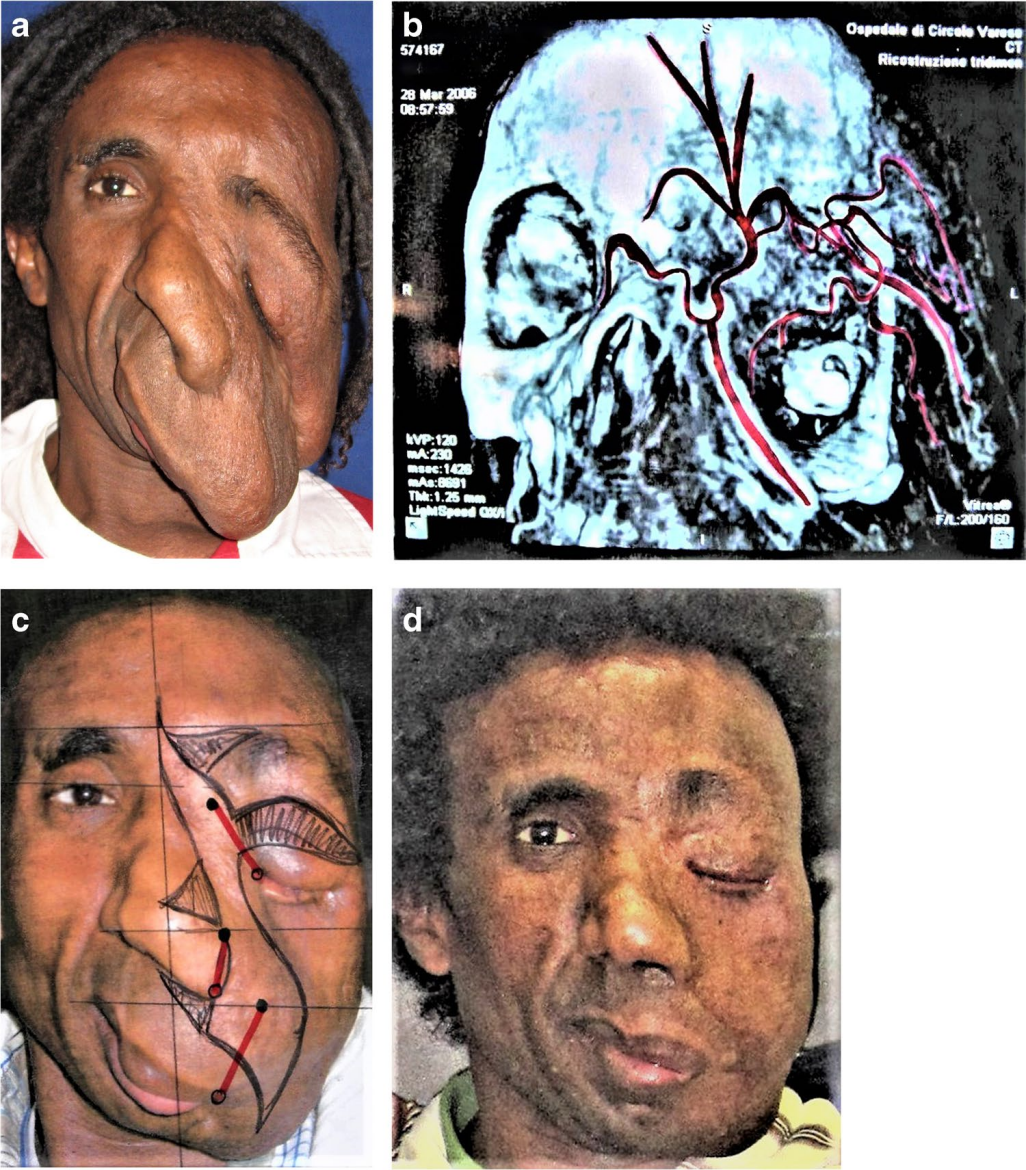
**A** A 29-year-old patient from Ethiopia with congenital neurofibromatosis of Recklinghausen (NF). **b** Arterial and venous extra-intracranial shunts through the orbit are typical routes of vascularization of the tumor, which are too wide for preoperative embolization. **c** Skin areas to be excised to lift up the cheek and fix it with Mitek anchors. Prior to excision, the main blood vessels were transcutaneously ligated. **d** After three operations, the patient happily returned to his home country and got married

**Fig. 5:**
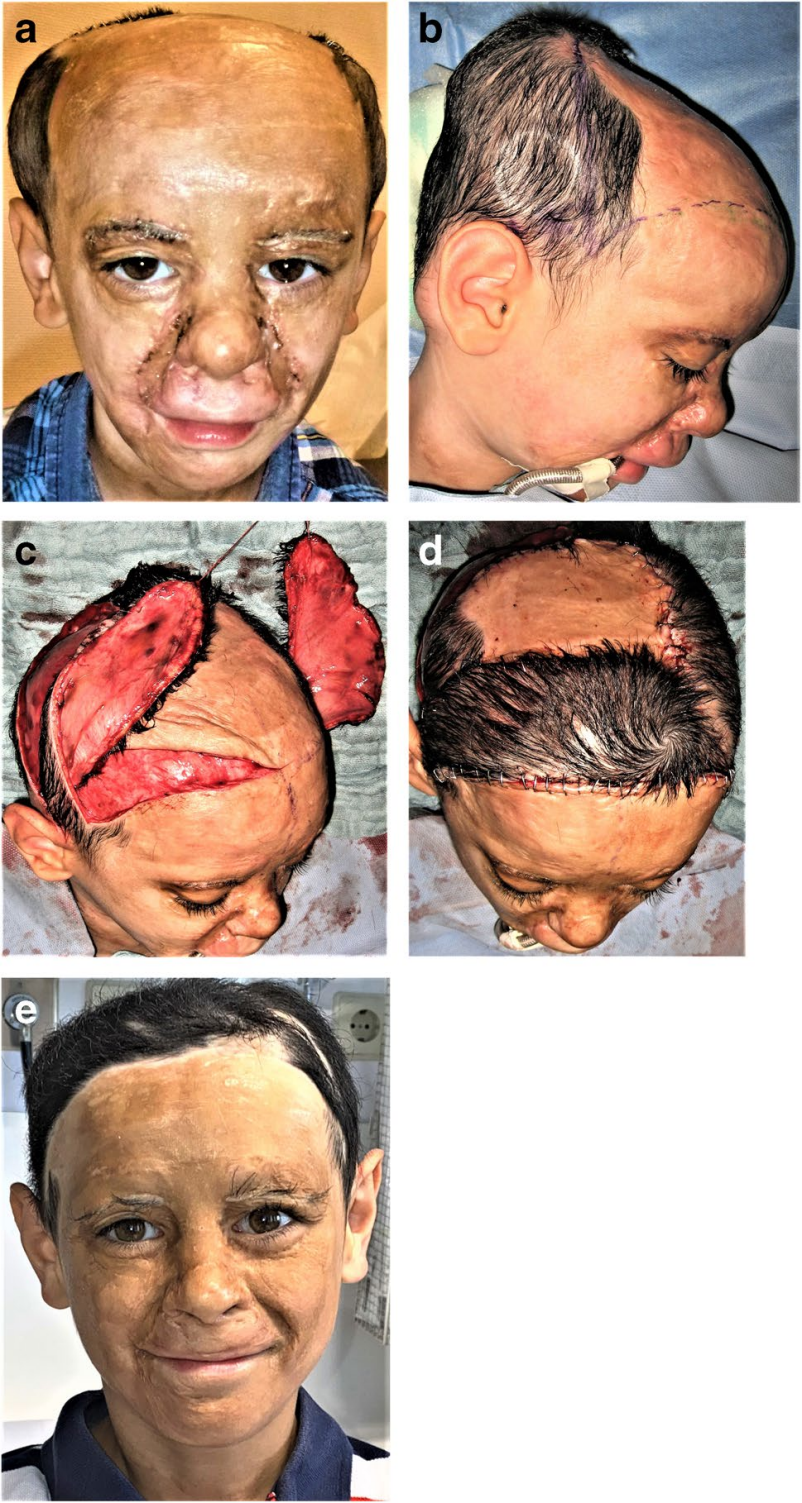
**A** This 8-year-old boy from Syria was ashamed of his scarred bald head which resulted from a severe burn [24]. **b** To reconstruct his scalp hair, two expanders were first placed under the remaining hair at the back of the head. **c** After 2 months of expansion, two swivel flaps stalked at the temporal artery (Juri flaps) were swung into the frontal hairline. **d** The parietal remaining large scar area was reduced with swivel flaps from a second expander under the hairline at the back of the head. **e** The boy is now radiant even without a cap

In Europe, after and between the operations, social accommodation with local families is necessary to avoid a possible culture shock. In addition, the return and reintegration in the home country after a few weeks or months in Germany is often difficult after the young patient and his/her mother have experienced our social system. A total of 160 patients with difficult and/or acute problems were taken to Bad Kreuznach for treatment in the past 20 years (Figs. 4a–d and 5a–d).

### Training of foreign colleagues in Europe

From the beginning, some INTERPLAST teams invited engaged interested colleagues from the local hospital to Frankfurt, Bad Kreuznach, and other INTERPLAST sections to learn the basics of plastic surgery in 3 months (more time our Foreigners Office did not permit).

Unfortunately, most colleagues from India or Africa did not return to their home country to help their countrymen but settled in the Gulf States—as “trained in Germany.” However, two exceptions must be mentioned here: Prof. Pius Agbenorku from Kumasi in Ghana established a department for plastic surgery at his university, which today invites students and young doctors from Germany. Dr. Prakash Chhajlani from Indore in India has accompanied every year one or two INTERPLAST teams worldwide since his visit to Frankfurt 40 years ago and today operates on patients with clefts or burn sequelae every Friday in his private clinic free of charge.

### The individual teacher in a developing country

Probably there are many more individual teachers who fly alone with a suitcase of instruments to colleagues or hospitals in developing countries to train and assist individual colleagues in surgery. For example, the recently deceased Belgian colleague Dr. Christian C. Dupuis [25] has been flying alone for many years to Provincial Hospitals to many countries of South East Asia like Vietnam, Laos, Cambodia, and Myanmar to teach 2 generations of general surgeons how to operate on clefts and burns. His average costs were around $1800.00 with using local assistants, nurses, and anesthetists, a tenth what an accompanying INTERPLAST team would have cost.

The chemistry between the inviting local surgeons and the visiting surgeon must be right, and when a working relationship is established, both feel committed to continue it. Sometimes, the relationship transforms itself into friendship to the benefit of the patients and local organizations like Lions or Rotary can be stimulated to support this local project.

Another colleague visited without a team from San Diego, CA, an interested general surgeon in a small hospital in El Salvador several times and introduced him to cleft surgery since there was no plastic surgeon far and wide in this endemic area of clefts. He left his special cleft instruments which were replicated overnight by instrument makers in Pakistan for a 10^th^ of the price of Western companies.

The author flew with his wife to Haiti in 2010 immediately after the earthquake to assist local general surgeons in caring for the mutilated victims. In the following missions, the INTERPLAST teams could already prepare for operations in simple tents as the earthquake had also destroyed all the hospitals in the region.

These teaching visits to engage local surgeons appear to be the most effective and sustainable way to local surgeons to become specialists and multipliers to guarantee certain plastic surgical operations in the future—and to make further INTERPLAST teams redundant. Another advantage is the training of the whole operating staff, the anesthetists, and the OR and ward nurses. And these lasting contacts facilitate the transport of difficult or time-consuming cases to plastic surgeons in their country or to Europe (Fig. 5a–d).

### The INTERPLAST Foundation and INTERPLAST hospitals

While a nonprofit organization (NGO) is required by law to use donations promptly, i.e., to implement them in actions within 2 years, a foundation, on the other hand, can accumulate endowment capital through tax-free “endowments”; the interest from which can be used to support projects regularly, even over many years.

With the financial backing of the INTERPLAST Foundation, four INTERPLAST hospitals have been founded and supported by INTERPLAST teams in the past. In 2004, the NGO INTERPLAST-Germany e.V. set up an INTERPLAST Foundation to support longer-term projects, such as equipping operation theaters and buying operating microscopes, anesthesia equipment and X-ray machines, power generators and solar panels, and even hospital ships in Myanmar.

The first INTERPLAST hospital was built in 1992 in Coroatà in the state of Maranhao, the poor district in northeastern Brazil, at the request of the bishop there. The hospital is regularly visited and filled with life by German-Brazilian mission teams. The humidity of the tropical climate made a total renovation urgently necessary recently.

The 2nd INTERPLAST hospital was founded in 1995 after the withdrawal of the Soviet troops in Jalalabad/Afghanistan, after 18 INTERPLAST teams had treated Afghan refugees in 2 hospitals in Peshawar/Pakistan since 1989. Among them were many children with torn off hands and legs by so-called “butterfly bombs,” which the Soviets had left in the fields. The cessation of financial aid by the EU—and the takeover of the hospital by the Taliban put an abrupt end to this extremely effective mission in 1999.

The 3rd Sushma Koirala Memorial Hospital (SKMH) 18 km east of Kathmandu in Nepal was established in 1997 to provide a common home for INTERPLAST teams from different countries. However, this did not succeed because of the independence of the national teams. On the other hand, it was possible to develop it into one of the most beautifully located and effective departments for plastic surgery with 50 beds and 5 plastic surgeons (www.nepalhospital.de). INTERPLAST-Germany and the SKM-Trust of the same name are currently trying to transform the hospital into a general hospital for the surrounding population and to link plastic surgery to the university in Kathmandu.

The 4th REHEMA (Swahili: mercy) Congo Hospital will be inaugurated in the spring of 2023 and will become a specialist hospital for the poor in the center of Goma, Democratic Republic of Congo (www.gomahospital.de). This hospital will also be connected to the newly established Faculty of Medicine of the University of Goma (UNIGOM) as soon as possible so that it can be taken over by the State in a few years.

### The modern virtual reality

The establishment of the Internet and the rapid spread of smart phones around the world, even to remote small towns in developing countries, allowed a previously unknown rapid communication with organizers of a prospective camp. Pictures of prospective patients can be sent in advance, and the team can prepare for them with special tools. Operation permits can be issued based on present physician documents, and customs authorities can be notified in advance of the team arriving with its precious materials.

After the return of the teams, the virtual contact regarding an optimal posttreatment, such as the cutting and repositioning of surgical flaps, is of great importance. If complications or new patients with difficult reconstructions arise, the INTERPLAST team leaders can give experienced advice based on photographs.

If necessary, even Zoom conferences can be held beforehand to get to know each other and to prepare the INTERPLAST team of unexpected eventualities. Many surgical methods can be viewed on YouTube or on medical portals so that it is not difficult for experienced surgeons to apply them in practice after consultation in Europe.

In Congo, for example, the training of 3 female doctors as general surgeons was financed through INTERPLAST-Germany: during their training, they continuously sent photos of patients with plastic surgery problems, which were promptly answered from Germany via e-mails with surgical drawings and articles—and were usually operated on satisfactorily.

Therefore, the future will be that local general surgeons or plastic surgeons will present their difficult cases in Zoom conferences and experts in Europe will prepare and discuss their surgery proposals. If we have left them dermatomes and skin mesher and they have performed some skin grafts with it, then they will be able to treat their burn patients optimally themselves with our virtual help in the future.

### European cooperation of individual INTERPLAST NGOs

Humanitarian aid in the field of plastic surgery should not only remain a national task but also a European idea to make collaboration possible and the help we can offer even more effective. During our *Annual INTERPLAST Symposium* in Bad Kreuznach, Germany, with international guests from France, Holland, Italy, and UK, we started 2004 to think about *INTERPLAST-Europe.* But all these ideas of international collaboration depend on the interest and input from all parties. If we share our experience and show more transparency of our activities to each other, then we will all benefit from this information and allow us to learn about problems and how to manage them better.

So, the idea of *SHARE* as the Committee on “Surgeons’ Humanitarian Aid Resource Europe” of the European Society of Plastic, Reconstructive and Aesthetic Surgery (ESPRAS) underlines the importance of collaboration of many other national charitable organizations. INTERPLAST-Germany will take active part in this process of coordination, improving communication and showing respect for other individuals engaged in humanitarian aid.

## Discussion

### Why is the humanitarian aid of INTERPLAST so effective?

Plastic surgery as a specialty requires almost no diagnostic effort in determining what to do or operate. This makes it ideal to establish “surgery camps” in medically underdeveloped locations. Local doctors can gather patients who require plastic surgery. After completed surgery, local surgeons or nurses can pull the sutures and send photographs to the team, should complications arise.

A decentralized organization structure allows that all activities are handled by the members who have organized the trip. All members are maximally motivated; they see their work as a mission; they donate their time during vacations or after retirement. Often singles or small groups of enthusiastic activists join these missions. The umbrella of the officially established INTERPLAST organization provides them with insurance and the financial framework [13].

We are currently experiencing a period of national self-assertion in many developing countries, which deliberately blocks or financially exploits humanitarian aid missions through bureaucratic hurdles. The new-found pride of these countries of not wanting to be dependent on foreign interventions often contrasts with the actual reality.

Unfortunately, government power and some cases of corruption stand in the way of our aid efforts and leave some teams disappointed and unwilling to further participate. These disappointing experiences, however, open new ways and possibilities to concentrate on single hospitals, which are willing to master the bureaucratic hurdles with their contacts to local customs.

**Fig. 6:**
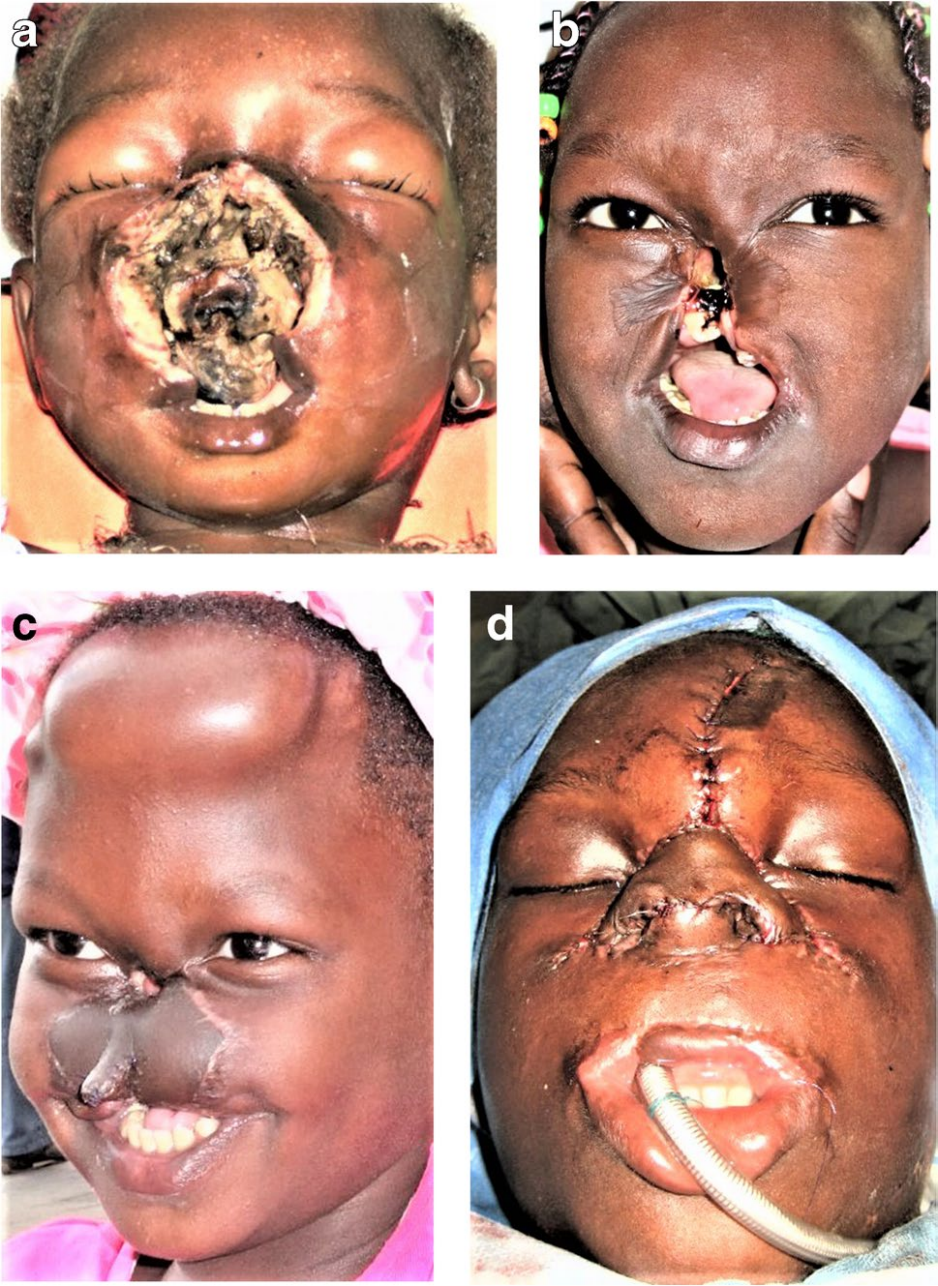
**A** This 8-year-old girl from Guinea-Bissau survived this necrotizing noma infection, whose causes are still unknown. **b** The sequelae required multistage reconstruction of bone, soft tissue, and skin. **c** A free osteocutaneous parascapular flap reconstructed the maxilla (OP Goetz Giessler) [26, 27]. **d** A three-layer nasal reconstruction with a pre-expanded forehead flap and airway opening perfected the reconstruction

**Fig. 7:**
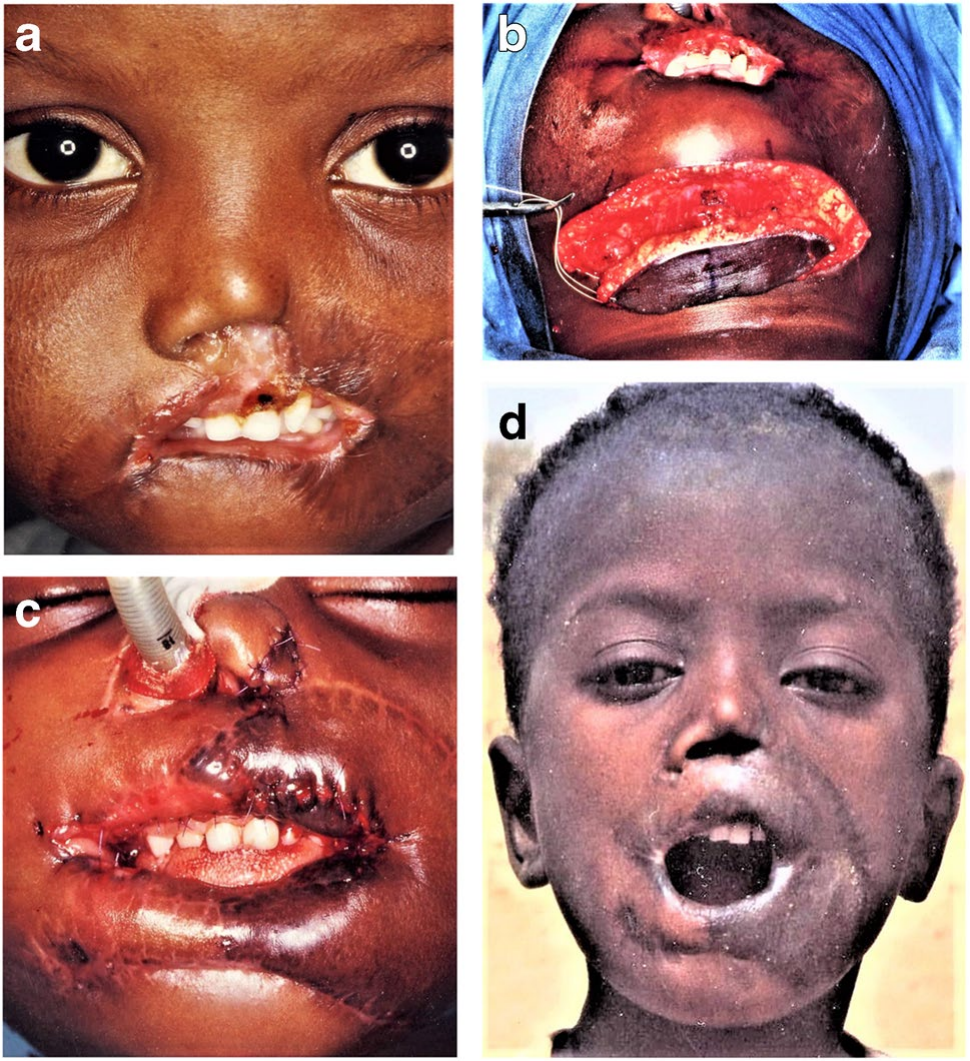
**A** This 4-year-old boy from Niger lost both lips after a noma infection. **b** The inner lining of the oral vestibule was provided by two overturning skin flaps, and for the outer lip reconstruction, a submental island flap was raised to cover vertically the area [28]. **c** Three weeks later, a vertical division of the flap was possible for the lower lip reconstruction. **d** Finally, the corners of the mouth were widened on both sides. **e** A few years later, the boy sent this picture with good lip function

In plastic surgery, some of the most challenging operations are facial defects after noma infections in infancy which caused high fever and localized necrosis of central parts of the face. Noma is caused by still unknown bacteria, possibly surviving on awns of millet and then multiplying explosively in the gingiva (Fig. 6a–d, necrosis). Often, the necrosis leads to bilateral constriction of temporomandibular joints during the healing period [26]. Eighty percent of infected children still die because no antibiotics are available. The surviving children are best be treated by free flaps to save the front from donor side (Fig. 7a–d, lis) [28].

**Fig. 8:**
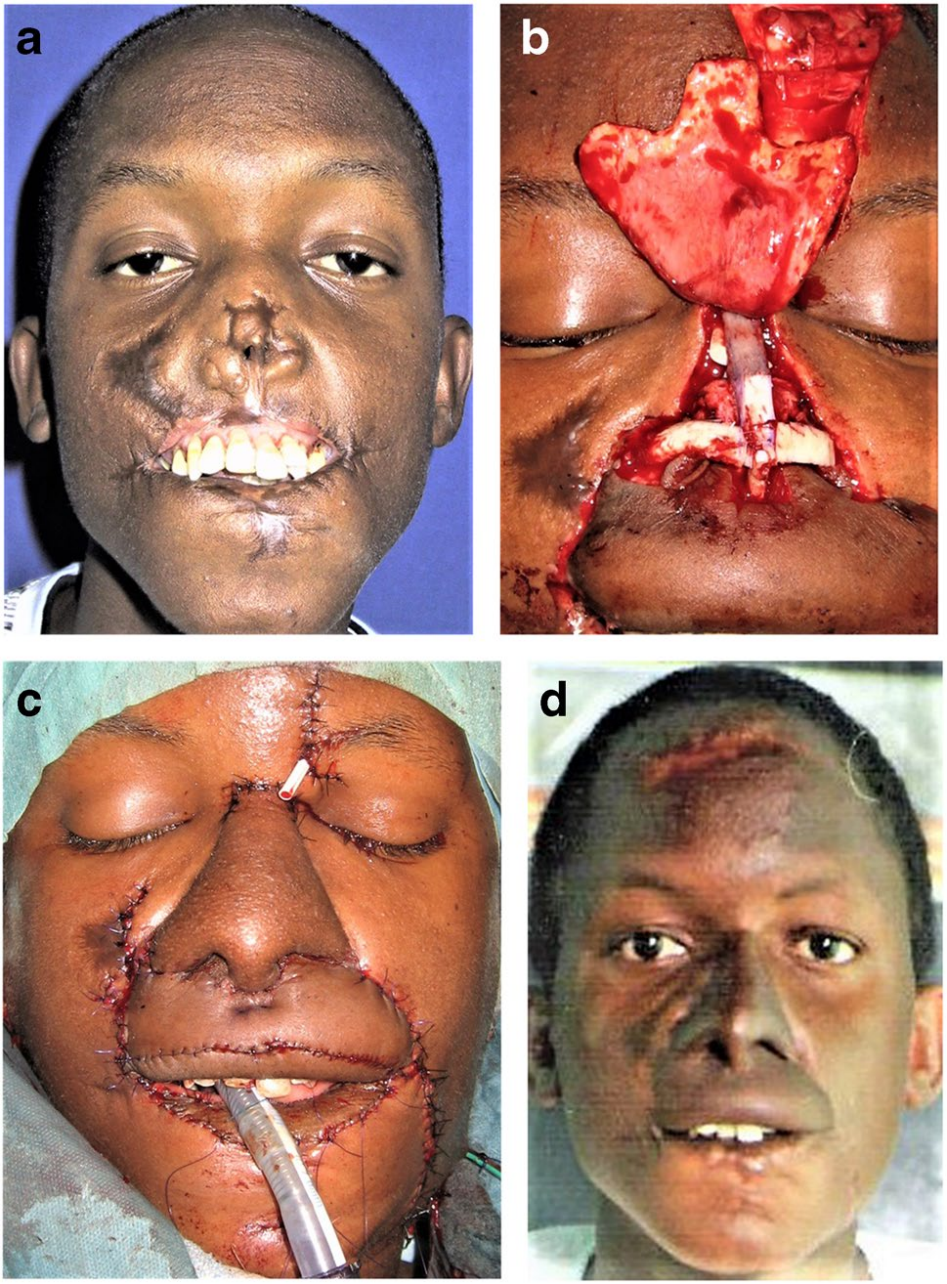
**A** A 21-year-old patient from Ethiopia with a central facial defect after burnt-out noma infection. **b** The lip defects were covered with a free radial flap and the nose with a paramedian forehead flap. **c** The inner lining of the nose was made by three local overturning flaps, and the tent-shaped nasal framework was carved from rib cartilage. **d** After final corrections to the lower lip, the patient returned to Ethiopia and sent us this picture

Whereas Resurge International teams consist of 10 to 20 members [21], INTERPLAST-Germany teams try to include as many local physicians, anesthetists, and nurses and therefore consist of only 4–8 team members. This reduced number of participants may be the reason one part of the difference in costs. A second reason may be the organization of patients and the postoperative care leave INTERPLAST-Germany teams to the physicians of the inviting local hospital. Similar to some ReSurge teams, some INTERPLAST-Germany teams are also led by or include microsurgeons, who do vascular anastomoses of free flaps in patients with noma sequelae with their surgical magnifiers (Fig. 8a–d) [27, 29].

## Conclusion and key points

### What we want to do in the host countries

INTERPLAST follows six steps of development aid:

1. *Mission*: pilot project on demand in order to find a good place to work where there is a real need of plastic surgery for social underprivileged people.
2. *Surgical camp*: if there is a good cooperation with the local team and the social partner is well organized, we can share our surgical experience for the benefit of the poor over years.
3. *Independence*: when teaching and assistance lead to a level of good quality of basic plastic surgery, we can reduce our support and remain available for expert questions.
4. *Political hurdles*: if too many hurdles in obtaining visa and at customs prevent flying teams, we try to invite individual surgeons to our congress and to observe plastic operations in Europe.
5. *Virtual contacts*: with today’s possibilities of the Internet, we can later assist them in difficult operations in their own operating room.
6. *Operations in Europe*: if the patients’ disfigurements are too difficult for our local colleagues, patients can be flown to Europe for treatment.

In the last years, we put emphasis on the *quality control* of our work and look for more *sustainability.* Camp registration and documentation became obligation for the team insurance and financial acceptance. In the new established *INTERPLAST Academy*, experienced plastic surgeons train our own team members in the special pathology of the developing countries and explain the basic plastic surgical technics suitable for the simple local facilities in the foreign environment. So, it will be much easier to perform *teaching* of the local doctors during the camp in order to enable them to help their own patients better.

### What we do not want to do in the host countries

We do not want to import neocolonialism, no self-producing performance, and no supermaterialism—we are behaving as guests, colleagues under colleagues and try to convince with team work in the surgical camp. We do not want to interfere with their social system although we try to support their needs. We do not want to create a dependency, because we respect their culture.

The success of a mission depends not only on the work the INTERPLAST team performs but mainly from the quality and responsibility of the host in the foreign country. We need an invitation, where help is needed (“mission on demand”) and never invite ourselves because of personal interest. Everybody can initiate a camp invitation, but we have to prove in advance how serious the invitation is and check the motivation of the host: charity, religious aspects, publicity, support for a local party, interest in medical and scientific exchange, or international cooperation.

We do not want to support persons who are only looking for their own profit while organizing a camp. We do not do cosmetic surgery and never take money for our operations in the foreign countries. The skills and risks of every operation should absolutely fit to the experience and quality of the surgeon; we do not do experimental surgery and do not use the trip only as training camp for our team members. There is no place for heroes and big adventure.

## Data Availability

The consent to publications of their photos is stored in their files in the Dieakonie Hospital in Bad Kreuznach
